# Associations between Serum Folate Concentrations and Functional Disability in Older Adults

**DOI:** 10.3390/antiox12030619

**Published:** 2023-03-02

**Authors:** Lujun Ji, Tianhao Zhang, Liming Zhang, Dongfeng Zhang

**Affiliations:** 1 Department of Epidemiology and Health Statistics, The School of Public Health of Qingdao University, 308 Ningxia Road, Qingdao 266071, China; 2 Department of Big Data in Health Science and Center for Clinical Big Data and Analytics, Second Affiliated Hospital and School of Public Health, Zhejiang University School of Medicine, Hangzhou 310058, China

**Keywords:** serum folate, functional disability, National Health and Nutrition Examination, older adults, dose–response relationship

## Abstract

Folate may have beneficial effects on physical function through its antioxidant effect. Thus, we investigated the associations between serum folate and functional disability in older adults. Data from the National Health and Nutrition Examination Survey 2011–2018 were used. Serum folate included 5-methyltetrahydrofolate and total folate. Five domains of functional disability, including lower extremity mobility (LEM), instrumental activities of daily living (IADL), activities of daily living (ADL), leisure and social activities (LSA), and general physical activities (GPA), were self-reported. Multivariable-adjusted logistic regression models and restricted cubic splines were employed. 5-Methyltetrahydrofolate was inversely associated with IADL and GPA disability, and the multivariate-adjusted ORs (95% CIs) in the highest versus lowest quartiles were 0.65 (0.46–0.91) and 0.70 (0.50–0.96), respectively. The total folate was also inversely associated with IADL (OR quartile 4vs1 = 0.65, 95% CI: 0.46–0.90) and GPA (OR quartile 3vs1 = 0.66, 95% CI: 0.44–0.99) disability. The dose–response relationships showed a gradual decrease in the risk of IADL and GPA disability as serum folate increased. In the sex, age, BMI, and alcohol consumption subgroup analyses, we saw that the associations were primarily found in females, under 80 years old, normal weight, and non-drinkers. Sensitivity analyses further confirmed the robustness of our results. Our results indicated that serum folate concentrations were negatively associated with IADL and GPA disability, especially in females. In other subgroup analyses, we discovered that these negative associations were primarily prevalent in participants under 80 years old, normal weight, and non-drinkers.

## 1. Introduction

Functional disability, defined as difficulty in performing basic activities of daily life [1], can induce a range of adverse health consequences, such as decreased quality of life [2], increased hospitalization rates [3], and increased mortality [4]. The number and proportion of older adults are increasing across many populations worldwide [5], and they tend to have a higher prevalence of functional disability [6], which imposes a heavy burden on society and families [7]. Consequently, exploring potentially modifiable factors that may prevent or delay the progression of functional disability is critical to reducing the burden on healthcare systems and achieving healthy aging.

Studies have shown that diet may be an effective way to prevent functional disability [8]. Recently, there has been considerable interest in the relationships between functional disability and dietary or nutritional factors, such as dietary protein [9], coffee [10], vitamin K [11], selenium [12], etc. Folate is a water-soluble vitamin that plays a role in the antioxidant process [13]. Folate can attenuate oxidative stress by improving biomarkers in the antioxidant defense system [13], and oxidative stress may increase the risk of disability [14]. Folate also acts as a coenzyme of one-carbon metabolism involved in homocysteine methylation, converting homocysteine to methionine [15,16]. Studies have shown that decreased homocysteine levels may be associated with improved physical function [17,18].

Some studies have investigated the relationship between dietary folate [19,20] or folate supplements [21] and physical function in older adults. Serum folate has also attracted the attention of a few researchers, because it can better reflect the recent folate intake level [22]. Still, there are few studies on the relationship between serum folate and physical function in older adults, and the results are inconsistent. To our knowledge, a study conducted in Singapore among 796 older participants discovered that a higher serum folate concentration was related to better physical performance on balance [23]. Another study of older adults in Spanish showed that people with a satisfactory folate status scored higher on the instrumental activities of daily living (IADL) test [24]. Nevertheless, a study of 698 older Italians found no significant relationship between serum folate and subsequent physical function (defined by the Short Physical Performance Battery (SPPB) test) [25].

5-Methyltetrahydrofolate (5-MTHF) is the main biologically active form of folate. Until recently, no studies have looked into the relationship between 5-MTHF and the risk of functional disability. Furthermore, disability is multidimensional, but previous studies have often been limited to one or two domains of functional disability. In addition, there was no clear dose–response relationship between serum folate and functional disability.

As a result, we extracted data from the National Health and Nutrition Examination Survey (NHANES) 2011–2018 cycles and performed this study to assess the associations between serum folate (including 5-MTHF and total folate) and five domains of functional disability in older Americans.

## 2. Materials and Methods

### 2.1. Study Population

The NHANES is a continuous survey utilizing a stratified multistage probability sample approach to assess the health and nutrition states of the United States (US)’ civilian people. The survey results are released publicly once every two years. The NHANES protocols were approved by the National Center for Health Statistics Ethics Review Committee, and each survey participant gave informed consent [26].

**Figure 1:**
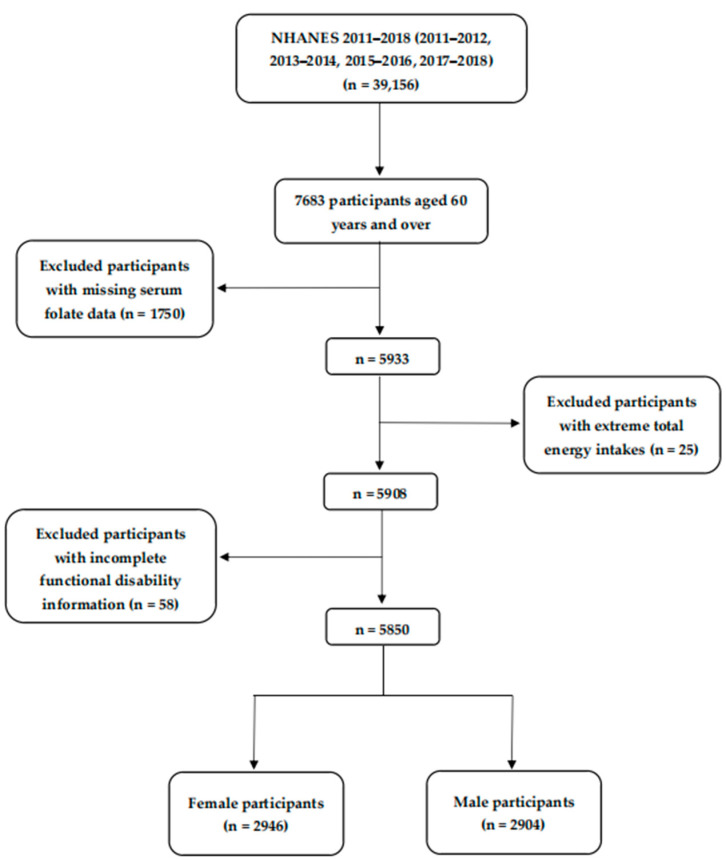
Flowchart of the screening process for the selection of eligible participants.

In this study, we combined four survey cycles of NHANES (2011–2012, 2013–1014, 2015–2016, and 2017–2018), totaling 39,156 individuals. Participants under the age of 60 were eliminated (*n* = 31,473). In addition, 1750 participants were ruled out because serum folate data were missing. Furthermore, participants with extreme total energy intakes (<500 or >5000 kcal/day for females, <500 or >8000 kcal/day for males) (*n* = 25) were removed [27]. After excluding 58 participants with incomplete functional disability information, leaving 5850 participants (2946 females and 2904 males) in the current study. Figure 1 depicts the specific screening procedure.

### 2.2. Serum Folate Assessment

Isotope dilution high-performance liquid chromatography coupled to tandem mass spectrometry (LC-MS/MS) was used to measure the concentrations of five bioactive forms of serum folate, including 5-MTHF, folic acid, 5-formyl-tetrahydrofolate, tetrahydrofolate, and 5,10-methenyl-tetrahydrofolate [28]. Five bioactive forms of serum folate were added together to estimate the total folate [29]. The main bioactive form among these five is 5-MTHF, which contributes about 90% of the total folate [30,31]. As a result, we included 5-MTHF and total folate in our investigations [32].

### 2.3. Self-Reported Functional Disability Assessment

The NHANES provided self-reported data on physical function [33]. Based on previous investigations [34,35,36,37], 19 well-validated questions of physical function were categorized into five domains of functional disability: lower extremity mobility (LEM), IADL, activities of daily living (ADL), general physical activities (GPA), and leisure and social activities (LSA). Detailed information is shown in Supplementary Table S1. Each question examined an individual’s ability to perform a task without using any special equipment. The options available for participants to answer were “no difficulty”, “some difficulty”, “much difficulty”, “unable to do”, or “do not do this activity”. Functional disability was defined as having any difficulty in performing one or more tasks within a given domain.

### 2.4. Other Covariates

Based on the prior literature [10,17], we took into account the influences of the following factors. The demographic factors included sex, age, race/ethnicity, educational level, marital status, and poverty–income ratio (PIR). We also adjusted the personal lifestyle factors such as total energy intake, physical activity, alcohol consumption, and smoking status. Furthermore, the health conditions factors included body mass index (BMI) and some chronic diseases, including hypertension, diabetes, arthritis, stroke, gout, cancer, congestive heart failure (CHF), coronary heart disease (CHD), angina, asthma, chronic bronchitis, and emphysema. Supplementary Table S2 presents the classifications of the covariates.

### 2.5. Statistical Analyses

We calculated the new sample weights in light of the NHANES weighting guidelines when merging the four 2-year cycles data to ensure the national representation of the sample. The Kolmogorov–Smirnov test was employed to identify the normality of the quantitative variables. We used the chi-square test to compare the differences between participants with and without functional disability for qualitative variables, the ANOVA test for normally distributed quantitative variables, and the Mann–Whitney *U* test for non-normally distributed quantitative variables.

In our analyses, considering the obvious differences in the serum folate levels between males and females and based on previous literature [32,38,39], we divided the serum folate data into four groups based on sex-specific quartiles, with the lowest quartile group serving as the reference group. Age-adjusted and multivariate-adjusted binary logistic regression analyses were conducted to evaluate the relationships between serum folate and all domains of functional disability. The multivariate-adjusted model adjusted for age, race/ethnicity, educational level, marital status, PIR, physical activity, alcohol consumption, smoking status, BMI, hypertension, diabetes, arthritis, stroke, gout, cancer, CHF, CHD, angina, asthma, chronic bronchitis, emphysema, and total energy intake. Sex, age, BMI, and alcohol consumption stratification analyses were also conducted in our study. To investigate the dose–response relationships between serum folate and functional disability, we employed restricted cubic splines with 3 knots (the 5th, 50th, and 95th percentiles of the serum folate distribution) in the multivariate-adjusted model.

To test the robustness of our results, we conducted the following sensitivity analyses. Firstly, considering that ignoring the presence of Mefox (an oxidation product of 5-MTHF) may lead to underestimation of the total folate [30], we further examined the relationships between the combined total folate (total folate plus Mefox) and all domains of functional disability. Secondly, we conducted secondary analyses by excluding participants using antibiotics, estrogens, and anticonvulsants. Thirdly, we minimized the confounding by excluding participants suffering from malnutrition. In addition, we additionally examined the relationships between the dietary folate intake and folate supplementation use (yes/no) and functional disability.

All statistical analyses were performed using Stata 15.0 and R software, version 4.2.1. Statistical significance was considered as two-sided *p*-values ≤ 0.05.

## 3. Results

### 3.1. Characteristics of the Participants

The characteristics of the participants are shown in Table 1 and Supplementary Table S3. For all domains of functional disability, people with a functional disability tended to be older; single; smokers; were more likely to have less total energy intake; lower PIR; higher BMI; a lower educational level; lower physical activity; and higher prevalence of stroke, hypertension, arthritis, diabetes, CHF, CHD, angina, asthma, chronic bronchitis, and emphysema. In addition to that, participants with functional disability were more likely to be females, except for ADL disability. Participants with disability in IADL, LEM, LSA, and ADL tended to be drinkers and minority races and were more likely to have lower serum folate concentrations. Supplementary Table S4 shows the comparison results of the serum folate concentrations between males and females. We found that females had higher serum folate concentrations than males.

### 3.2. Relationships between Serum Folate and Functional Disability

Table 2 and Supplementary Table S5 display the weighted odds ratios (ORs) with 95% confidence intervals (CIs) for all domains of functional disability according to the quartiles of 5-MTHF and total folate. In the age-adjusted models, the concentrations of 5-MTHF and total folate were inversely related to all domains of functional disability. In the multivariate-adjusted models, we found negative relationships between 5-MTHF and IADL and GPA disability, the ORs (95% CIs) in the highest versus lowest quartiles were 0.65 (0.46–0.91) and 0.70 (0.50–0.96), respectively. We also found that elevated level of total folate was associated with decreased odds of disability in IADL (OR quartile 4vs1 = 0.65, 95% CI: 0.46–0.90) and GPA (OR quartile 3vs1 = 0.66, 95% CI: 0.44–0.99).

We further performed stratified analyses by sex. For females, after adjusted age, we observed that 5-MTHF and total folate were negatively associated with the odds of all domains of functional disability. Compared with quartile 1 (Q1), the fully adjusted ORs (95% CIs) for IADL disability for the highest quartile of 5-MTHF and total folate were 0.52 (0.35–0.79) and 0.53 (0.35–0.78), respectively. In the multivariate-adjusted models, the risk of GPA disability decreased in Q2–Q4 for 5-MTHF and total folate (adjusted ORs ranged from 0.46 to 0.56). The results are shown in Table 3 and Supplementary Table S6. However, no statistical significance was found in males in the multivariate-adjusted models (Supplementary Tables S7 and S8).

**Figure 2:**
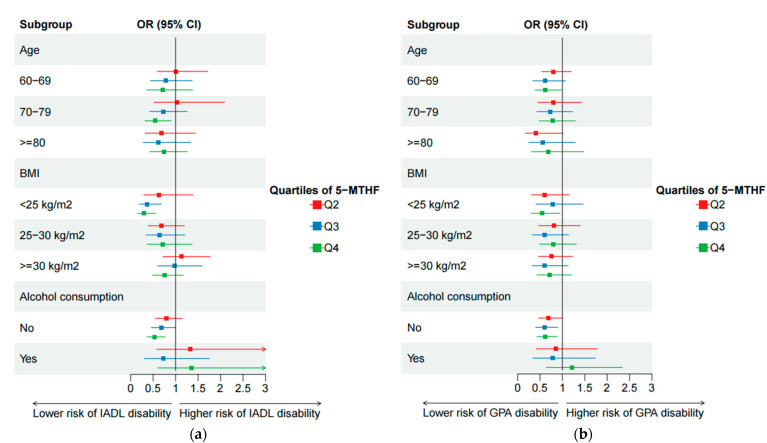
Subgroup analyses of the relationships (multivariate-adjusted odds ratios and 95% confidence intervals) between 5-Methyltetrahydrofolate and the risk of IADL disability (**a**) and GPA disability (**b**).

Figure 2 presents the associations between 5-MTHF and the risk of IADL and GPA disability in stratified analyses by age, BMI, and alcohol consumption. Among people aged 70–79 years old, normal weight, and non-drinkers, 5-MTHF was negatively associated with IADL disability. In addition, we found that 5-MTHF was negatively associated with GPA disability in the population of 60–69 years old, normal weight, and non-drinkers. The stratified analyses of total folate and the risk of IADL and GPA disability yielded similar findings (Supplementary Figure S1).

**Figure 3:**
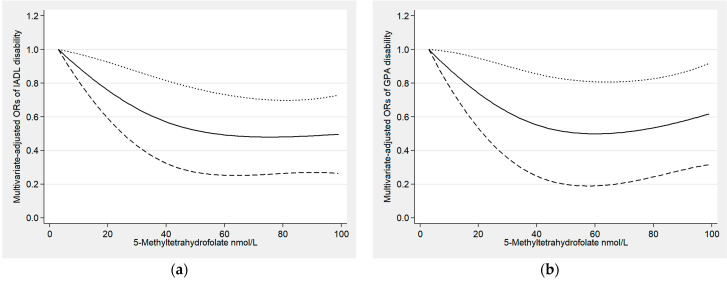

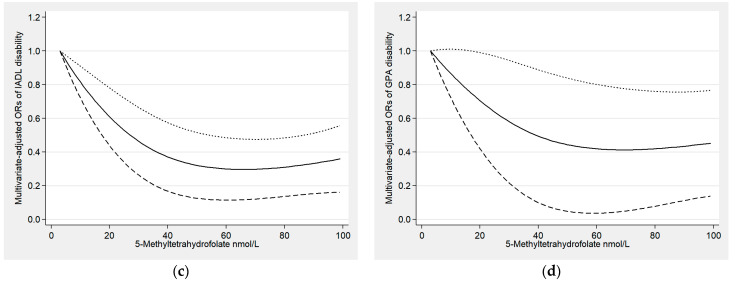
(**a**) Examination of the dose–response relationship between 5-Methyltetrahydrofolate and IADL disability (*p* for non-linearity = 0.131); (**b**) examination of the dose–response relationship between 5-Methyltetrahydrofolate and GPA disability (*p* for non-linearity = 0.083); (**c**) examination of the dose–response relationship between 5-Methyltetrahydrofolate and IADL disability in females (*p* for non-linearity = 0.015); (**d**) examination of the dose–response relationship between 5-Methyltetrahydrofolate and GPA disability in females (*p* for non-linearity = 0.21). The solid line and dashed lines represent the estimated odds ratios and the 95% confidence intervals.

In Figure 3, the outcomes of the restricted cubic spline analyses between 5-MTHF and the risk of IADL and GPA disability are displayed. Among overall participants and females, we found that as 5-MTHF levels increased, the risk of IADL and GPA disability decreased gradually. We found similar dose–response relationships between total folate and the risk of IADL and GPA disability (Supplementary Figure S2).

### 3.3. Sensitivity Analyses

The results of the sensitivity analyses of the associations between combined total folate and all domains of functional disability were consistent with our primary results (Supplementary Table S9). After excluding 798 participants using antibiotics, estrogens, and anticonvulsants, 5-MTHF and total folate were negatively associated with three domains of functional disability (IADL, LSA, and GPA) (Supplementary Table S10). After eliminating 192 participants suffering from malnutrition, the results did not change substantially (Supplementary Table S11). Supplementary Table S12 presents the relationships between dietary folate intake and the risk of functional disability. Compared with Q1, the multivariate-adjusted ORs (95% CIs) for IADL disability of dietary folate intake in the Q3 and Q4 were 0.68 (0.50–0.93) and 0.62 (0.45–0.86), respectively. The associations between folate supplementation use and functional disability risk are shown in Supplementary Table S13. Compared with non-supplement users (*n* = 2168), folate supplement users had a lower prevalence of ADL disability, with an OR (95% CI) of 0.78 (0.64–0.96).

## 4. Discussion

In this cross-sectional study of 5850 participants, after adjusting all covariates, higher concentrations of serum folate were related to lower odds of disability in IADL and GPA. In sex-stratified analyses, we found that serum folate concentrations were adversely associated with IADL and GPA disability in females, but no such associations were found in males. In age, BMI, and alcohol consumption subgroup analyses, negative associations between serum folate with the risk of IADL disability were observed in the population of 70–79 years old, normal weight, and non-drinkers, and with the risk of GPA disability were mainly observed in the population of 60–69 years old, normal weight, and non-drinkers. The dose–response relationships showed a gradual decrease in the risk of IADL and GPA disability as serum folate increased. Sensitivity analyses further confirmed the robustness of our results.

An observational study involving 796 Singapore older adults aged 55 and above indicated that relatively higher serum folate concentration was related to better performance in the balance test [23]. Another survey of Spanish older adults aged 65 to 89 found that participants who performed better on the IADL test had higher serum folate concentrations than those who performed worse [24]. In addition, a review summarized the existing evidence that some vitamin deficiencies had a negative impact on the functional recovery of older adults, including folate [40]. These studies provide indirect support for our findings. It is worth mentioning that several studies have found that dietary folate and folate supplement intakes may be partially associated with improved physical function in older adults [20,21], which further supported our findings. Nevertheless, a 3-year cohort study of 698 older adults showed that serum folate was not associated with subsequent SPPB test scores [25]. The inconsistent findings may be due to the differences in the specific items covered by the test for assessing physical function.

The mechanisms underlying the link between serum folate and functional disability remained unclear, and there may be the following aspects. Firstly, folate may have beneficial effects on physical function through its antioxidant effect [41,42]. Several studies have shown that folate or folic acid supplementation may attenuate oxidative stress by improving biomarkers in the antioxidative defense system, such as increased serum total antioxidant capacity and glutathione (GSH) concentration [13,43,44,45,46]. Studies have found that oxidative stress is an independent predictor of functional disability [14]. Secondly, the anti-inflammatory effect of folate [47] may also be a mechanism for decreasing the risk of functional disability [48,49]. In addition, folate as a coenzyme of one-carbon metabolism involved homocysteine methylation and promotes homocysteine to methionine conversion [50,51,52]. Several studies have suggested that elevated homocysteine levels may be a risk factor for functional disability [17] or physical function decline [18,53,54]. Specifically, homocysteine may impair physical function through mechanisms such as increasing the concentration of reactive oxygen species and reducing the bioavailability of nitric oxide [55].

In the results of sex stratification, we only found negative associations between serum folate and the risk of functional disability in females. The first possible reason may be that the improvement of serum folate on biomarkers in the antioxidant defense system varies by gender. A meta-analysis found that short-term folate supplementation significantly increased serum GSH concentration in females, but this effect was not observed in males [13]. Second, Larry A. Tucker analyzed data from NHANES and suggested that women with low folate levels were more prone to have telomere shortening and cell aging, but no such association was found in men [56]. An animal experiment showed that transplanting aging cells into young mice could cause continuous physical dysfunction [57]. Another possible reason for the significant gender differences may be as stated above. Third, many studies have shown that homocysteine levels tend to be higher in males than in females [58,59,60]. In addition to folate, homocysteine levels are influenced by other factors, such as sex hormones [61,62]. Finally, sex differences in folate metabolism may also be a possible cause [63]. Additionally, other subgroup analyses found that the associations of higher serum folate with decreased risk of disability in IADL and GPA were mainly in the population under 80 years old, normal weight, and non-drinkers. To our knowledge, several studies have suggested that age and obesity were possible risk factors for functional disability [64,65,66]. Therefore, we speculate that the detrimental effects of age and obesity might counteract the beneficial effects of folate on functional disability. In addition, studies have shown that alcohol consumption may interfere with the absorption and action of folate [67,68], which may partly attenuate the beneficial effects of folate. Further studies are necessary to understand the possible biological mechanisms underlying subgroup differences in this association.

It is worth noting that studies have shown that poor diet and poor appetite are the main causes of folate deficiency [69,70], Poor absorption owing to gastrointestinal dysfunction/disease can also result in folate deficiency [71,72]. The physiological process of aging makes older adults more susceptible to these nutritional problems, such as poor taste, loss of appetite, and gastrointestinal malabsorption [73]. Older adults tend to have a higher prevalence of folate deficiency [74]. In addition, poor diet and malabsorption may cause or contribute to the deterioration of physical, intellectual, or mental function and then generate a series of functional impairments and even disabilities [75]. Therefore, we recommend appropriately increasing the intake of folate-rich foods, especially in the older population with poor diet and gastrointestinal dysfunction/disease.

The present study has several advantages. First, we conducted the study using NHANES data, which employs a complex sampling design and stringent quality control to obtain representative samples of American residents, lending credibility to our findings. Second, the relationship between 5-MTHF (the primary biological activity form of serum folate) and functional disability was examined in our study for the first time. Third, we evaluated multiple domains of functional disability. In addition, we further explored the dose–response relationships between serum folate concentrations and functional disability. However, we must acknowledge that our research has the following flaws. First, this study was a cross-sectional study, and causality cannot be inferred. Second, although we referred to previous studies [10,35,37] and used 19 well-validated questions of physical function to define functional disability, but functional disability was defined by self-reported questionnaires rather than objective measurements of physical function, which may lead to recall bias and influence the accuracy of participants’ physical function status. While some studies have shown a high correlation between self-reported physical function and objective physical function measures [76,77]. Third, although we evaluated as many different domains of functional disability as possible, there are still some types of disability that are not considered, such as vision loss and hearing loss. Fourth, although we carefully adjusted the potential confounding factors, residual confounding may still exist. In addition, considering that the use of a single indicator, such as serum creatinine, is insufficient to diagnose kidney disease [78], our study did not exclude participants with kidney dysfunction/disease. Since kidney function strongly affected homocysteine levels [79], participants with kidney dysfunction/disease may have influenced the results. Finally, our study focused on older adults aged 60 and over in the US. Given the disparities in serum folate levels and functional disability prevalence rates across countries and ages, the current findings should be applied with caution to other age and region groups.

## 5. Conclusions

Our results indicated that serum folate concentrations were negatively associated with IADL and GPA disability, especially in females. In other subgroup analyses, we found that these negative associations primarily occurred in participants under 80 years old, normal weight, and non-drinkers. Further longitudinal studies and biological mechanism research should be conducted to confirm our findings.

## Figures and Tables

**Table 1:** Characteristics of participants by IADL and GPA disability, NHANES 2011–2018 (*n* = 5850).

Characteristics	With IADL Disability	Without IADL Disability	*p* Value	With GPA Disability	Without GPA Disability	*p* Value
Number of participants, *n* (%)	1703 (29.11)	4147 (70.89)		3699 (63.23)	2151 (36.77)	
Age (year), *n* (%) ^a^			<0.001			<0.001
60–69	764 (42.37)	2269 (57.89)		1717 (47.32)	1316 (64.98)	
70–79	502 (31.94)	1220 (28.81)		1125 (31.79)	597 (25.91)	
≥80	437 (25.69)	658 (13.29)		857 (20.89)	238 (9.11)	
Sex, *n* (%) ^a^			<0.001			<0.001
Female	1002 (65.70)	1944 (50.29)		2054 (59.94)	892 (44.74)	
Male	701 (34.30)	2203 (49.71)		1645 (40.06)	1259 (55.26)	
Race/ethnicity, *n* (%) ^a^			<0.001			0.898
Mexican American	218 (5.25)	464 (3.77)		422 (4.17)	260 (4.16)	
Other Hispanic	192 (4.80)	447 (3.61)		400 (4.10)	239 (3.63)	
Non-Hispanic White	741 (71.10)	1833 (78.18)		1732 (76.21)	842 (76.45)	
Non-Hispanic Black	382 (10.64)	899 (7.84)		768 (8.57)	513 (8.61)	
Other races	170 (8.21)	504 (6.59)		377 (6.95)	297 (7.16)	
Educational level, *n* (%) ^a^			<0.001			<0.001
Below high school	593 (22.44)	1039 (13.75)		1124 (18.52)	508 (11.78)	
High school	412 (27.71)	934 (23.35)		896 (25.94)	450 (22.02)	
Above high school	693 (49.85)	2170 (62.91)		1671 (55.54)	1192 (66.20)	
Marital status, *n* (%) ^a^			<0.001			<0.001
Not living alone	785 (52.76)	2538 (66.79)		1913 (58.33)	1410 (71.29)	
Living alone	916 (47.24)	1605 (33.21)		1781 (41.67)	740 (28.71)	
Poverty–income ratio, *n* (%) ^a^			<0.001			<0.001
<1	402 (14.91)	596 (7.43)		731 (11.45)	267 (5.89)	
≥1	1301 (85.09)	3551 (92.57)		2968 (88.55)	1884 (94.11)	
Physical activity, *n* (%) ^a^			<0.001			<0.001
Low	1149 (63.24)	1927 (41.81)		2244 (54.80)	832 (34.78)	
High	540 (36.76)	2205 (58.19)		1433 (45.20)	1312 (65.22)	
Body mass index, *n* (%) ^a^			<0.001			<0.001
<25 kg/m^2^	371 (21.73)	1086 (24.08)		785 (19.79)	672 (29.77)	
25 to <30 kg/m^2^	491 (30.30)	1579 (38.68)		1208 (33.55)	862 (41.57)	
≥30 kg/m^2^	769 (47.98)	1445 (37.24)		1608 (46.66)	606 (28.67)	
Smoking status, *n* (%) ^a^	899 (53.44)	2008 (48.43)	0.043	1931 (51.79)	976 (46.24)	0.007
Alcohol consumption, *n* (%) ^a^	281 (20.23)	554 (15.01)	0.011	584 (17.52)	251 (14.31)	0.077
Hypertension, *n* (%) ^a^	1448 (83.98)	3241 (74.40)	<0.001	3058 (79.81)	1631 (71.97)	<0.001
Diabetes, *n* (%) ^a^	602 (27.50)	1002 (19.88)	<0.001	1162 (24.95)	442 (16.61)	<0.001
Arthritis, *n* (%) ^a^	1151 (72.22)	1753 (45.62)	<0.001	2286 (64.25)	618 (32.57)	<0.001
Stroke, *n* (%) ^a^	263 (13.09)	218 (4.83)	<0.001	392 (9.07)	89 (3.46)	<0.001
Gout, *n* (%) ^a^	209 (12.12)	340 (8.99)	0.005	407 (10.72)	142 (8.26)	0.068
Cancer, *n* (%) ^a^	373 (27.26)	811 (24.29)	0.154	827 (27.28)	357 (21.25)	0.001
Congestive heart failure, *n* (%) ^a^	230 (10.59)	202 (4.64)	<0.001	372 (7.93)	60 (3.25)	<0.001
Coronary heart disease, *n* (%) ^a^	245 (14.33)	339 (9.10)	<0.001	443 (11.91)	141 (8.02)	0.007
Angina, *n* (%) ^a^	145 (8.76)	171 (4.32)	0.002	260 (7.02)	56 (2.86)	0.002
Asthma, *n* (%) ^a^	326 (20.44)	489 (12.24)	<0.001	615 (16.82)	200 (10.23)	<0.001
Chronic bronchitis, *n* (%) ^a^	214 (14.60)	215 (6.57)	<0.001	369 (11.54)	60 (3.76)	<0.001
Emphysema, *n* (%) ^a^	125 (8.67)	109 (2.80)	<0.001	200 (5.93)	34 (1.63)	<0.001
Total energy intake (kcal/day), median (IQR) ^b^	1670.8 (835.5)	1765 (858.5)	<0.001	1702.5 (832)	1800.5 (877)	<0.001
Total folate (nmol/L), median (IQR) ^b^	41.8 (40.7)	45.4 (39.6)	0.002	44.1 (41.1)	45.1 (37.8)	0.412
5-Methyltetrahydrofolate (nmol/L), median (IQR) ^b^	39.2 (38)	43.1 (37.7)	<0.001	41.2 (38.9)	42.6 (35.9)	0.227

Data are the number of participants (weighted percentage) or medians (interquartile ranges). IADL, instrumental activities of daily living; GPA, general physical activities. ^a^ Chi-square test was used to compare the percentage between participants with and without functional disability. ^b^ Mann–Whitney *U* test was used to compare the difference between participants with and without functional disability.

**Table 2:** Weighted odds ratios (95% confidence intervals) for all domains of functional disability across quartiles of 5-Methyltetrahydrofolate.

	Quartile of 5-Methyltetrahydrofolate			
	Q1	Q2	Q3	Q4
LEM				
Cases/Participants	709/1469	596/1465	552/1456	584/1460
Age-adjusted	Ref.	0.72 (0.59–0.89) **	0.58 (0.46–0.73) **	0.62 (0.51–0.74) **
Multivariate-adjusted	Ref.	1.05 (0.78–1.43)	0.80 (0.57–1.14)	0.87 (0.65–1.16)
IADL				
Cases/Participants	494/1469	437/1465	373/1456	399/1460
Age-adjusted	Ref.	0.77 (0.59–0.99) *	0.60 (0.45–0.79) **	0.56 (0.42–0.74) **
Multivariate-adjusted	Ref.	0.92 (0.63–1.33)	0.74 (0.52–1.04)	0.65 (0.46–0.91) *
ADL				
Cases/Participants	448/1469	400/1465	350/1456	354/1460
Age-adjusted	Ref.	0.79 (0.58–1.06)	0.71 (0.53–0.95) *	0.70 (0.55–0.90) **
Multivariate-adjusted	Ref.	1.00 (0.69–1.45)	0.90 (0.61–1.32)	0.87 (0.62–1.21)
LSA				
Cases/Participants	421/1469	361/1465	329/1456	319/1460
Age-adjusted	Ref.	0.75 (0.57–1.00)	0.69 (0.53–0.90) **	0.59 (0.43–0.82) **
Multivariate-adjusted	Ref.	0.93 (0.64–1.35)	0.87 (0.60–1.25)	0.74 (0.48–1.14)
GPA				
Cases/Participants	979/1469	918/1465	881/1456	921/1460
Age-adjusted	Ref.	0.72 (0.58–0.89) **	0.64 (0.50–0.81) **	0.64 (0.50–0.82) **
Multivariate-adjusted	Ref.	0.77 (0.56–1.05)	0.66 (0.44–0.99) *	0.70 (0.50–0.96) *

Calculated using binary logistic regression models. Q, quartile; LEM, lower extremity mobility; IADL, instrumental activities of daily living; ADL, activities of daily living; LSA, leisure and social activities; GPA, general physical activities; PIR, poverty–income ratio; BMI, body mass index. The multivariate-adjusted model adjusted for age, race/ethnicity, educational level, marital status, PIR, physical activity, alcohol consumption, smoking status, BMI, hypertension, diabetes, arthritis, stroke, gout, cancer, congestive heart failure, coronary heart disease, angina, asthma, chronic bronchitis, emphysema, and total energy intake. Q1: <28.2 nmol/L for females and <25.7 nmol/L for males; Q2: 28.2–45.0 nmol/L for females and 25.7–38.7 nmol/L for males; Q3: 45.0–70.9 nmol/L for females and 38.7–58.9 nmol/L for males; Q4: ≥70.9 nmol/L for females and ≥58.9 nmol/L for males. * *p* < 0.05; ** *p* < 0.01.

**Table 3:** Weighted odds ratios (95% confidence intervals) for all domains of functional disability across quartiles of 5-Methyltetrahydrofolate in females.

	Quartile of 5-Methyltetrahydrofolate			
	Q1	Q2	Q3	Q4
LEM				
Cases/Participants	425/737	341/742	310/732	326/735
Age-adjusted	Ref.	0.75 (0.54–1.03)	0.58 (0.42–0.80) **	0.49 (0.37–0.65) **
Multivariate-adjusted	Ref.	0.97 (0.58–1.62)	0.78 (0.45–1.36)	0.63 (0.40–1.00)
IADL				
Cases/Participants	313/737	249/742	205/732	235/735
Age-adjusted	Ref.	0.62 (0.47–0.81) **	0.44 (0.31–0.61) **	0.45 (0.33–0.63) **
Multivariate-adjusted	Ref.	0.73 (0.47–1.14)	0.49 (0.32–0.74) **	0.52 (0.35–0.79) **
ADL				
Cases/Participants	253/737	208/742	175/732	184/735
Age-adjusted	Ref.	0.70 (0.47–1.03)	0.61 (0.42–0.88) **	0.68 (0.46–0.99) *
Multivariate-adjusted	Ref.	0.87 (0.52–1.45)	0.64 (0.37–1.10)	0.79 (0.44–1.42)
LSA				
Cases/Participants	245/737	202/742	173/732	178/735
Age-adjusted	Ref.	0.67 (0.47–0.95) *	0.60 (0.42–0.87) **	0.56 (0.39–0.81) **
Multivariate-adjusted	Ref.	0.76 (0.47–1.25)	0.68 (0.39–1.19)	0.68 (0.41–1.14)
GPA				
Cases/Participants	575/737	507/742	479/732	493/735
Age-adjusted	Ref.	0.50 (0.35–0.73) **	0.49 (0.35–0.69) **	0.43 (0.29–0.62) **
Multivariate-adjusted	Ref.	0.53 (0.32–0.88) *	0.56 (0.33–0.96) *	0.51 (0.32–0.79) **

Calculated using binary logistic regression models. The multivariate-adjusted model adjusted for age, race/ethnicity, educational level, marital status, PIR, physical activity, alcohol consumption, smoking status, BMI, hypertension, diabetes, arthritis, stroke, gout, cancer, congestive heart failure, coronary heart disease, angina, asthma, chronic bronchitis, emphysema, and total energy intake. Q1: <28.2 nmol/L; Q2: 28.2–45.0 nmol/L; Q3: 45.0–70.9 nmol/L; Q4: ≥70.9 nmol/L. * *p* < 0.05; ** *p* < 0.01.

## Data Availability

The raw data that support the findings of this study are available from the National Health and Nutrition Examination Survey: https://www.cdc.gov/nchs/nhanes/index.htm (accessed on 10 November 2022).

## Supplementary Materials

The following supporting information can be downloaded at: https://www.mdpi.com/article/10.3390/antiox12030619/s1, Figure S1. Subgroup analyses of the relationships (multivariate-adjusted odds ratios and 95% confidence intervals) between total folate and the risk of IADL disability (a) and GPA disability (b); Figure S2. (a) Examination of the dose–response relationship between total folate and IADL disability (*p* for non-linearity = 0.296); (b) examination of the dose–response relationship between total folate and GPA disability (*p* for non-linearity = 0.154); (c) examination of the dose–response relationship between total folate and IADL disability in females (*p* for non-linearity = 0.047); (d) examination of the dose–response relationship between total folate and GPA disability in females (*p* for non-linearity = 0.345). The solid line and dashed lines represent the estimated odds ratios and the 95% confidence intervals; Table S1. Questions about self-reported functional disability; Table S2. The classifications of covariates; Table S3. Characteristics of participants by LEM, ADL, and LSA disability, NHANES 2011–2018 (*n* = 5850); Table S4. Comparison of several serum folate concentrations by sex; Table S5. Weighted odds ratios (95% confidence intervals) for all domains of functional disability across quartiles of total folate; Table S6. Weighted odds ratios (95% confidence intervals) for all domains of functional disability across quartiles of total folate in females; Table S7. Weighted odds ratios (95% confidence intervals) for all domains of functional disability across quartiles of 5-Methyltetrahydrofolate in males; Table S8. Weighted odds ratios (95% confidence intervals) for all domains of functional disability across quartiles of total folate in males; Table S9. Sensitivity analyses of weighted odds ratios (95% confidence intervals) for functional disability across quartiles of combined total folate; Table S10. Sensitivity analyses of weighted odds ratios (95% confidence intervals) for functional disability across quartiles of serum folate after excluding participants using antibiotics, estrogens, and anticonvulsants; Table S11. Sensitivity analyses of weighted odds ratios (95% confidence intervals) for functional disability across quartiles of serum folate after excluding participants suffering from malnutrition [80]; Table S12. Sensitivity analyses of weighted odds ratios (95% confidence intervals) for functional disability across quartiles of dietary folate intake; Table S13. Sensitivity analyses of the association between folate supplement users and non-supplement users with functional disability.
