# Supplementary material for: Associations between Serum Folate Concentrations and Functional Disability in Older Adults
Source: Antioxidants (Basel). 2023 Mar 2;12(3):619. doi: 10.3390/antiox12030619 (PMC10045063; doi:10.3390/antiox12030619)
Supplement: Supplementary file 1 [file antioxidants-12-00619-s001.zip › Supplementary.pdf]

Table S1. Questions about self-reported functional disability.

Functional disability domains	Questions [a]
Lower extremity mobility (LEM)	
	Walking for a quarter mile
	Walking up 10 steps
Instrumental activities of daily living (IADL)	
	Managing money
	Doing chores around the house
	Preparing meals
Activities of daily living (ADL)	
	Eating
	Dressing
	Getting in and out of bed
	Walking between rooms on the same floor
Leisure and social activities (LSA)	
	Going out to shopping, movies, or events
	Doing leisure activities at home
	Participating in social activities
General physical activities (GPA)	
	Stooping/crouching/kneeling
	Standing on feet for about 2 hours
	Sitting for about 2 hours
	Standing up from an armless chair
	Reaching up overhead
	Using fingers to grasp/handle small objects
	Lifting or carrying 10 pounds

[a] Participants were asked:" By yourself and without using any special equipment, how much difficulty do you have …?"

Table S2. The classifications of covariates.

Covariates	Classifications
Age	60–69; 70–79; ≥ 80
Sex	Male; Female
Race/Ethnicity	Mexican American; Other Hispanic; Non-Hispanic White; Non-Hispanic Black; Other races
Educational level	Below high school; High school; Above high school
Marital status	Not living alone; Living alone
Poverty-income ratio (PIR)	< 1; ≥ 1
Physical activity [a]	Low; High
Body mass index (BMI)	< 25 kg/m^2; 25 to < 30 kg/m^2; ≥ 30 kg/m^2
Smoking status	Yes (Smoked at least 100 cigarettes in life); No
Alcohol consumption	Yes (Had 4/5 or more drinks every day); No
Total energy intake (kcal/day) [b]	Continuous
Hypertension [c]	Yes; No
Diabetes [d]	Yes; No
Arthritis [e]	Yes; No
Stroke [e]	Yes; No
Gout [e]	Yes; No
Cancer [e]	Yes; No
Congestive heart failure [e]	Yes; No
Coronary heart disease [e]	Yes; No
Angina [e]	Yes; No
Asthma [e]	Yes; No
Chronic bronchitis [e]	Yes; No
Emphysema [e]	Yes; No

[a] Physical activity was calculated and classified into two categories: "low (< 600 MET minutes/week)" and "high (≥ 600 MET-minutes/week)" according to the Global Physical Activity Questionnaire analysis guide.

[b] Total energy intake of each participant was the average of two 24-hour dietary energy intake.

[c] Hypertension was defined as mean systolic blood pressure ≥ 130 mmHg, or mean diastolic blood pressure ≥ 80 mmHg, or ever been told have hypertension, or taking prescribed medicine now.

[d] Diabetes was defined as self-reported physician diagnosis, or taking insulin, or taking anti-diabetic pills.

[e] Arthritis, stroke, gout, cancer, congestive heart failure, coronary heart disease, angina, asthma, chronic bronchitis, and emphysema were defined as if self-reported by a physician diagnosis.

Table S3. Characteristics of participants by LEM, ADL, and LSA disability, NHANES 2011–2018 (n = 5850).

Characteristics	With LEM disability	Without LEM disability	*p* value	With ADL disability	Without ADL disability	*p* value	With LSA disability	Without LSA disability	*p* value
Number of participants, n (%)	2441 (41.73)	3409 (58.27)		1552 (26.53)	4298 (73.47)		1430 (24.44)	4420 (75.56)	
Age (year), n (%) [a]			<0.001			<0.001			<0.001
60–69	1041 (40.81)	1992 (61.91)		703 (43.53)	2330 (56.73)		627 (43.16)	2406 (56.61)	
70–79	747 (33.35)	975 (27.32)		445 (29.89)	1277 (29.57)		434 (31.20)	1288 (29.23)	
≥ 80	653 (25.85)	442 (10.77)		404 (26.57)	691 (13.70)		369 (25.64)	726 (14.16)	
Sex, n (%) [a]			<0.001			0.067			<0.001
Female	1402 (62.93)	1544 (49.03)		820 (57.86)	2126 (53.38)		798 (62.07)	2148 (52.33)	
Male	1039 (37.07)	1865 (50.97)		732 (42.14)	2172 (46.62)		632 (37.93)	2272 (47.67)	
Race/ethnicity, n (%) [a]			0.002			<0.001			<0.001
Mexican American	265 (4.35)	417 (4.05)		207 (5.94)	475 (3.65)		196 (5.98)	486 (3.68)	
Other Hispanic	237 (3.80)	402 (4.01)		190 (5.50)	449 (3.47)		153 (4.74)	486 (3.71)	
Non-Hispanic White	1130 (73.69)	1444 (77.93)		634 (68.13)	1940 (78.66)		620 (69.76)	1954 (78.05)	
Non-Hispanic Black	578 (10.51)	703 (7.38)		365 (11.72)	916 (7.68)		325 (11.12)	956 (7.91)	
Other races	231 (7.65)	443 (6.63)		156 (8.71)	518 (6.54)		136 (8.39)	538 (6.66)	
Educational level, n (%) [a]			<0.001			<0.001			<0.001
Below high school	799 (21.20)	833 (12.83)		554 (23.21)	1078 (13.98)		501 (22.16)	1131 (14.42)	
High school	608 (28.55)	738 (21.97)		370 (26.39)	976 (23.96)		361 (29.50)	985 (23.17)	
Above high school	1027 (50.25)	1836 (65.20)		623 (50.40)	2240 (62.06)		564 (48.34)	2299 (62.41)	
Marital status, n (%) [a]			<0.001			<0.001			<0.001
Not living alone	1142 (53.21)	2181 (69.24)		740 (54.29)	2583 (65.60)		676 (50.96)	2647 (66.31)	
Living alone	1294 (46.79)	1227 (30.76)		810 (45.71)	1711 (34.40)		754 (49.04)	1767 (33.69)	
Poverty-income ratio, n (%) [a]			<0.001			<0.001			<0.001
< 1	551 (14.70)	447 (6.10)		390 (15.77)	608 (7.58)		375 (17.47)	623 (7.26)	
≥ 1	1890 (85.30)	2962 (93.90)		1162 (84.23)	3690 (92.42)		1055 (82.53)	3797 (92.74)	
Physical activity, n (%) [a]			<0.001			<0.001			<0.001
Low	1674 (65.90)	1402 (35.99)		1056 (65.89)	2020 (42.17)		995 (66.36)	2081 (42.44)	
High	747 (34.10)	1998 (64.01)		483 (34.11)	2262 (57.83)		424 (33.64)	2321 (57.56)	
Body mass index, n (%) [a]			<0.001			<0.001			<0.001
< 25 kg/m^2	466 (19.20)	991 (26.06)		287 (18.21)	1170 (24.92)		275 (18.38)	1182 (24.79)	
25 to < 30 kg/m^2	710 (29.18)	1360 (40.96)		471 (32.03)	1599 (37.75)		425 (31.46)	1645 (37.82)	
≥ 30 kg/m^2	1173 (51.62)	1041 (32.98)		717 (49.76)	1497 (37.33)		664 (50.17)	1550 (37.40)	
Smoking status, n (%) [a]	1316 (53.75)	1591 (47.26)	0.003	836 (54.67)	2071 (48.34)	0.011	780 (55.12)	2127 (48.33)	0.002
Alcohol consumption, n (%) [a]	404 (19.11)	431 (14.69)	0.014	282 (24.02)	553 (14.24)	<0.001	242 (19.79)	593 (15.45)	0.045
Hypertension, n (%) [a]	2082 (82.45)	2607 (73.50)	<0.001	1323 (83.91)	3366 (74.93)	<0.001	1246 (86.06)	3443 (74.51)	<0.001
Diabetes, n (%) [a]	842 (29.06)	762 (17.41)	<0.001	559 (29.94)	1045 (19.58)	<0.001	502 (29.47)	1102 (19.88)	<0.001
Arthritis, n (%) [a]	1636 (68.42)	1268 (42.83)	<0.001	1094 (71.70)	1810 (47.17)	<0.001	999 (72.47)	1905 (47.39)	<0.001
Stroke, n (%) [a]	347 (12.61)	134 (3.52)	<0.001	255 (14.95)	226 (4.73)	<0.001	232 (14.28)	249 (5.08)	<0.001
Gout, n (%) [a]	300 (10.98)	249 (9.10)	0.175	204 (13.22)	345 (8.84)	<0.001	179 (12.23)	370 (9.18)	0.005
Cancer, n (%) [a]	549 (27.35)	635 (23.66)	0.089	341 (27.76)	843 (24.30)	0.081	308 (27.36)	876 (24.47)	0.132
Congestive heart failure, n (%) [a]	331 (11.77)	101 (2.75)	<0.001	211 (10.99)	221 (4.84)	<0.001	198 (11.21)	234 (4.89)	<0.001
Coronary heart disease, n (%) [a]	359 (16.45)	225 (6.77)	<0.001	208 (12.95)	376 (9.77)	0.035	210 (14.45)	374 (9.43)	0.004
Angina, n (%) [a]	210 (10.09)	106 (2.63)	<0.001	132 (9.47)	184 (4.35)	<0.001	134 (9.82)	182 (4.35)	<0.001
Asthma, n (%) [a]	467 (19.63)	348 (11.14)	<0.001	300 (21.46)	515 (12.37)	<0.001	285 (21.32)	530 (12.57)	<0.001
Chronic bronchitis, n (%) [a]	291 (14.09)	138 (5.32)	<0.001	177 (13.12)	252 (7.42)	<0.001	175 (14.11)	254 (7.26)	<0.001
Emphysema, n (%) [a]	170 (7.91)	64 (2.13)	<0.001	102 (8.37)	132 (3.21)	<0.001	114 (9.57)	120 (2.98)	<0.001
Total energy intake (kcal/day), median (IQR) [b]	1678.5 (819.5)	1786 (878.5)	<0.001	1686.5 (866.5)	1754 (848)	0.007	1683.5 (842.5)	1753 (856.5)	<0.001
Total folate (nmol/L), median (IQR) [b]	42.3 (41.6)	45.8 (38.6)	<0.001	41.8 (39.2)	45.4 (40.2)	<0.001	41.7 (39.5)	45.4 (40)	<0.001
5-Methyltetrahydrofolate (nmol/L), median (IQR) [b]	39.5 (39.5)	43.3 (36.4)	<0.001	38.9 (37)	43 (38.3)	<0.001	38.9 (37.6)	42.8 (38)	<0.001

Data are the number of participants (weighted percentage) or medians (interquartile ranges). [a] Chi-square test was used to compare the percentage between participants with and without functional disability. [b] Mann–Whitney *U* test was used to compare the difference between participants with and without functional disability.

Table S4. Comparison of several serum folate concentrations by sex.

	Females	Males	*p* value
5-Methyltetrahydrofolate	45.0 (28.2, 70.9)	38.7 (25.7, 58.9)	<0.001
Total folate	47.8 (30.5, 75.3)	41.2 (27.9, 62.5)	<0.001
Combined total folate	50.3 (32.9, 77.7)	43.7 (29.9, 65.3)	<0.001

Data are presented with medians (P25, P75).

Table S5. Weighted odds ratios (95% confidence intervals) for all domains of functional disability across quartiles of total folate.

	Quartile of total folate			
	Q1	Q2	Q3	Q4
LEM				
Cases/Participants	696/1467	607/1465	546/1460	592/1458
Age-adjusted	Ref.	0.76 (0.62–0.94) *	0.60 (0.47–0.77) **	0.66 (0.55–0.80) **
Multivariate-adjusted	Ref.	1.03 (0.77–1.39)	0.82 (0.57–1.19)	0.93 (0.71–1.23)
IADL				
Cases/Participants	483/1467	442/1465	379/1460	399/1458
Age-adjusted	Ref.	0.78 (0.61–1.00)	0.65 (0.50–0.85) **	0.57 (0.43–0.75) **
Multivariate-adjusted	Ref.	0.88 (0.62–1.24)	0.80 (0.57–1.13)	0.65 (0.46–0.90) *
ADL				
Cases/Participants	434/1467	402/1465	359/1460	357/1458
Age-adjusted	Ref.	0.81 (0.61–1.08)	0.80 (0.60–1.06)	0.73 (0.56–0.94) *
Multivariate-adjusted	Ref.	0.97 (0.68–1.39)	1.07 (0.73–1.57)	0.86 (0.61–1.20)
LSA				
Cases/Participants	414/1467	367/1465	323/1460	326/1458
Age-adjusted	Ref.	0.75 (0.57–0.99) *	0.69 (0.53–0.90) **	0.62 (0.44–0.86) **
Multivariate-adjusted	Ref.	0.82 (0.58–1.17)	0.88 (0.61–1.27)	0.75 (0.49–1.13)
GPA				
Cases/Participants	974/1467	918/1465	875/1460	932/1458
Age-adjusted	Ref.	0.70 (0.56–0.87) **	0.64 (0.50–0.83) **	0.67 (0.52–0.86) **
Multivariate-adjusted	Ref.	0.70 (0.51–0.97) *	0.66 (0.44–0.99) *	0.72 (0.52–1.00)

Calculated using binary logistic regression models. Q, quartile. The multivariate-adjusted model adjusted for age, race/ethnicity, educational level, marital status, PIR, physical activity, alcohol consumption, smoking status, BMI, hypertension, diabetes, arthritis, stroke, gout, cancer, congestive heart failure, coronary heart disease, angina, asthma, chronic bronchitis, emphysema, and total energy intake. Q1: <30.5 nmol/L for females and <27.9 nmol/L for males; Q2: 30.5–47.8 nmol/L for females and 27.9–41.2 nmol/L for males; Q3: 47.8–75.3 nmol/L for females and 41.2–62.5 nmol/L for males; Q4: ≥75.3 nmol/L for females and ≥62.5 nmol/L for males. * $p < 0.05$; ** $p < 0.01$.

Table S6. Weighted odds ratios (95% confidence intervals) for all domains of functional disability across quartiles of total folate in females.

	**Quartile of total folate**			
	**Q1**	**Q2**	**Q3**	**Q4**
LEM				
Cases/Participants	416/737	349/737	306/739	331/733
Age-adjusted	Ref.	0.82 (0.58–1.16)	0.58 (0.41–0.83) **	0.55 (0.41–0.73) **
Multivariate-adjusted	Ref.	1.02 (0.61–1.72)	0.81 (0.45–1.44)	0.72 (0.46–1.12)
IADL				
Cases/Participants	303/737	253/737	212/739	234/733
Age-adjusted	Ref.	0.65 (0.50–0.86) **	0.49 (0.36–0.67) **	0.47 (0.34–0.65) **
Multivariate-adjusted	Ref.	0.71 (0.46–1.12)	0.55 (0.38–0.81) **	0.53 (0.35–0.78) **
ADL				
Cases/Participants	244/737	209/737	180/739	187/733
Age-adjusted	Ref.	0.73 (0.51–1.07)	0.68 (0.48–0.98) *	0.69 (0.47–1.02)
Multivariate-adjusted	Ref.	0.84 (0.50–1.41)	0.77 (0.45–1.33)	0.75 (0.41–1.36)
LSA				
Cases/Participants	240/737	206/737	167/739	185/733
Age-adjusted	Ref.	0.70 (0.50–0.98) *	0.58 (0.40–0.86) **	0.61 (0.43–0.87) **
Multivariate-adjusted	Ref.	0.74 (0.47–1.17)	0.68 (0.38–1.20)	0.73 (0.45–1.16)
GPA				
Cases/Participants	572/737	504/737	483/739	495/733
Age-adjusted	Ref.	0.48 (0.33–0.70) **	0.49 (0.34–0.70) **	0.44 (0.30–0.63) **
Multivariate-adjusted	Ref.	0.46 (0.27–0.77) **	0.54 (0.30–0.96) *	0.50 (0.32–0.77) **

Calculated using binary logistic regression models. The multivariate-adjusted model adjusted for age, race/ethnicity, educational level, marital status, PIR, physical activity, alcohol consumption, smoking status, BMI, hypertension, diabetes, arthritis, stroke, gout, cancer, congestive heart failure, coronary heart disease, angina, asthma, chronic bronchitis, emphysema, and total energy intake. Q1: <30.5 nmol/L; Q2: 30.5–47.8 nmol/L; Q3: 47.8–75.3 nmol/L; Q4: ≥75.3 nmol/L. * $p < 0.05$; ** $p < 0.01$.

Table S7. Weighted odds ratios (95% confidence intervals) for all domains of functional disability across quartiles of 5-Methyltetrahydrofolate in males.

	**Quartile of 5-Methyltetrahydrofolate**			
	**Q1**	**Q2**	**Q3**	**Q4**
LEM				
Cases/Participants	284/732	255/723	242/724	258/725
Age-adjusted	Ref.	0.65 (0.45–0.96) *	0.61 (0.42–0.90) *	0.85 (0.59–1.23)
Multivariate-adjusted	Ref.	1.17 (0.74–1.85)	0.91 (0.58–1.41)	1.34 (0.78–2.32)
IADL				
Cases/Participants	181/732	188/723	168/724	164/725
Age-adjusted	Ref.	1.07 (0.71–1.62)	1.02 (0.65–1.59)	0.80 (0.53–1.21)
Multivariate-adjusted	Ref.	1.52 (0.79–2.92)	1.55 (0.93–2.60)	1.06 (0.58–1.92)
ADL				
Cases/Participants	195/732	192/723	175/724	170/725
Age-adjusted	Ref.	0.92 (0.63–1.34)	0.86 (0.54–1.38)	0.74 (0.52–1.06)
Multivariate-adjusted	Ref.	1.30 (0.82–2.08)	1.29 (0.76–2.18)	1.01 (0.65–1.58)
LSA				
Cases/Participants	176/732	159/723	156/724	141/725
Age-adjusted	Ref.	0.91 (0.61–1.34)	0.86 (0.60–1.24)	0.66 (0.43–1.01)
Multivariate-adjusted	Ref.	1.39 (0.78–2.48)	1.31 (0.91–1.89)	0.86 (0.48–1.53)
GPA				
Cases/Participants	404/732	411/723	402/724	428/725
Age-adjusted	Ref.	0.97 (0.65–1.44)	0.82 (0.54–1.24)	0.96 (0.67–1.38)
Multivariate-adjusted	Ref.	1.08 (0.65–1.80)	0.72 (0.42–1.23)	0.92 (0.57–1.47)

Calculated using binary logistic regression models. The multivariate-adjusted model adjusted for age, race/ethnicity, educational level, marital status, PIR, physical activity, alcohol consumption, smoking status, BMI, hypertension, diabetes, arthritis, stroke, gout, cancer, congestive heart failure, coronary heart disease, angina, asthma, chronic bronchitis, emphysema, and total energy intake. Q1: <25.7 nmol/L; Q2: 25.7–38.7 nmol/L; Q3: 38.7–58.9 nmol/L; Q4: ≥58.9 nmol/L. * $p < 0.05$.

Table S8. Weighted odds ratios (95% confidence intervals) for all domains of functional disability across quartiles of total folate in males.

	Quartile of total folate			
	Q1	Q2	Q3	Q4
LEM				
Cases/Participants	280/730	258/728	240/721	261/725
Age-adjusted	Ref.	0.67 (0.46–0.96) *	0.66 (0.45–0.95) *	0.89 (0.62–1.26)
Multivariate-adjusted	Ref.	1.09 (0.74–1.61)	0.93 (0.60–1.45)	1.36 (0.80–2.31)
IADL				
Cases/Participants	180/730	189/728	167/721	165/725
Age-adjusted	Ref.	1.05 (0.70–1.58)	1.07 (0.69–1.65)	0.81 (0.53–1.24)
Multivariate-adjusted	Ref.	1.41 (0.79–2.52)	1.60 (0.95–2.69)	1.06 (0.58–1.92)
ADL				
Cases/Participants	190/730	193/728	179/721	170/725
Age-adjusted	Ref.	0.94 (0.66–1.35)	0.98 (0.63–1.54)	0.78 (0.54–1.14)
Multivariate-adjusted	Ref.	1.31 (0.82–2.10)	1.50 (0.87–2.59)	1.08 (0.67–1.73)
LSA				
Cases/Participants	174/730	161/728	156/721	141/725
Age-adjusted	Ref.	0.83 (0.57–1.22)	0.90 (0.63–1.29)	0.66 (0.42–1.01)
Multivariate-adjusted	Ref.	1.09 (0.62–1.91)	1.29 (0.88–1.88)	0.81 (0.45–1.45)
GPA				
Cases/Participants	402/730	414/728	392/721	437/725
Age-adjusted	Ref.	0.97 (0.66–1.43)	0.86 (0.57–1.31)	1.04 (0.72–1.49)
Multivariate-adjusted	Ref.	1.03 (0.63–1.69)	0.75 (0.45–1.26)	0.98 (0.61–1.58)

Calculated using binary logistic regression models. The multivariate-adjusted model adjusted for age, race/ethnicity, educational level, marital status, PIR, physical activity, alcohol consumption, smoking status, BMI, hypertension, diabetes, arthritis, stroke, gout, cancer, congestive heart failure, coronary heart disease, angina, asthma, chronic bronchitis, emphysema, and total energy intake. Q1: <27.9 nmol/L; Q2: 27.9–41.2 nmol/L; Q3: 41.2–62.5 nmol/L; Q4: ≥62.5 nmol/L. * $p < 0.05$.

Table S9. Sensitivity analyses of weighted odds ratios (95% confidence intervals) for functional disability across quartiles of combined total folate.

	Quartile of combined total folate			
	**Q1**	**Q2**	**Q3**	**Q4**
LEM	Ref.	1.10 (0.80–1.50)	0.88 (0.60–1.31)	0.97 (0.72–1.31)
IADL	Ref.	1.00 (0.70–1.44)	0.81 (0.57–1.17)	0.69 (0.51–0.95) *
ADL	Ref.	1.03 (0.71–1.50)	1.08 (0.73–1.60)	0.89 (0.63–1.26)
LSA	Ref.	0.81 (0.57–1.16)	0.87 (0.61–1.25)	0.75 (0.50–1.13)
GPA	Ref.	0.70 (0.51–0.97) *	0.70 (0.47–1.04)	0.68 (0.49–0.95) *

Calculated using binary logistic regression models. Model adjusted for age, race/ethnicity, educational level, marital status, PIR, physical activity, alcohol consumption, smoking status, BMI, hypertension, diabetes, arthritis, stroke, gout, cancer, congestive heart failure, coronary heart disease, angina, asthma, chronic bronchitis, emphysema, and total energy intake. Q1: <32.9 nmol/L for females and <29.9 nmol/L for males; Q2: 32.9–50.3 nmol/L for females and 29.9–43.7 nmol/L for males; Q3: 50.3–77.7 nmol/L for females and 43.7–65.3 nmol/L for males; Q4: ≥77.7 nmol/L for females and ≥65.3 nmol/L for males. * $p < 0.05$.

Table S10. Sensitivity analyses of weighted odds ratios (95% confidence intervals) for functional disability across quartiles of serum folate after excluding participants using antibiotics, estrogens, and anticonvulsants.

	Quartile of serum folate concentrations			
	**Q1**	**Q2**	**Q3**	**Q4**
5-Methyltetrahydrofolate (nmol/L)				
LEM	Ref.	1.01 (0.75–1.36)	0.81 (0.55–1.19)	0.99 (0.69–1.41)
IADL	Ref.	0.90 (0.60–1.36)	0.77 (0.53–1.11)	0.66 (0.45–0.95) *
ADL	Ref.	0.89 (0.57–1.38)	0.85 (0.57–1.28)	0.78 (0.55–1.10)
LSA	Ref.	0.82 (0.54–1.23)	0.85 (0.58–1.25)	0.61 (0.37–1.00) *
GPA	Ref.	0.71 (0.50–1.02)	0.68 (0.44–1.06)	0.70 (0.49–0.99) *
Total folate (nmol/L)				
LEM	Ref.	1.05 (0.79–1.40)	0.86 (0.59–1.27)	1.07 (0.75–1.51)
IADL	Ref.	0.82 (0.57–1.17)	0.83 (0.58–1.20)	0.63 (0.45–0.89) **
ADL	Ref.	0.88 (0.58–1.34)	1.00 (0.66–1.52)	0.80 (0.56–1.14)
LSA	Ref.	0.75 (0.52–1.10)	0.91 (0.61–1.36)	0.62 (0.39–0.99) *
GPA	Ref.	0.64 (0.44–0.91) *	0.67 (0.44–1.03)	0.71 (0.50–1.00) *

Calculated using binary logistic regression models. Model adjusted for age, race/ethnicity, educational level, marital status, PIR, physical activity, alcohol consumption, smoking status, BMI, hypertension, diabetes, arthritis, stroke, gout, cancer, congestive heart failure, coronary heart disease, angina, asthma, chronic bronchitis, emphysema, and total energy intake. 5-Methyltetrahydrofolate: Q1: <28.5 nmol/L for females and <26.1 nmol/L for males; Q2: 28.5–45.0 nmol/L for females and 26.1–38.9 nmol/L for males; Q3: 45.0–70.8 nmol/L for females and 38.9–58.9 nmol/L for males; Q4: ≥70.8 nmol/L for females and ≥58.9 nmol/L for males. Total folate: Q1: <30.6 nmol/L for females and <28.1 nmol/L for males; Q2: 30.6–47.7 nmol/L for females and 28.1–41.4 nmol/L for males; Q3: 47.7–75.0 nmol/L for females and 41.4–62.5 nmol/L for males; Q4: ≥75.0 nmol/L for females and ≥62.5 nmol/L for males. * $p < 0.05$; ** $p < 0.01$.

Table S11. Sensitivity analyses of weighted odds ratios (95% confidence intervals) for functional disability across quartiles of serum folate after excluding participants suffering from malnutrition [a].

	Quartile of serum folate concentrations			
	Q1	Q2	Q3	Q4
5-Methyltetrahydrofolate (nmol/L)				
LEM	Ref.	1.04 (0.77–1.41)	0.82 (0.58–1.17)	0.86 (0.64–1.14)
IADL	Ref.	0.94 (0.64–1.38)	0.74 (0.53–1.04)	0.62 (0.43–0.88) **
ADL	Ref.	1.03 (0.70–1.53)	0.97 (0.66–1.43)	0.83 (0.58–1.20)
LSA	Ref.	0.93 (0.62–1.39)	0.92 (0.62–1.35)	0.72 (0.45–1.16)
GPA	Ref.	0.76 (0.56–1.04)	0.67 (0.44–1.02)	0.69 (0.50–0.96) *
Total folate (nmol/L)				
LEM	Ref.	1.09 (0.81–1.46)	0.83 (0.56–1.22)	0.98 (0.74–1.30)
IADL	Ref.	0.92 (0.64–1.32)	0.78 (0.55–1.11)	0.66 (0.47–0.92) *
ADL	Ref.	1.01 (0.70–1.45)	1.06 (0.72–1.57)	0.85 (0.59–1.24)
LSA	Ref.	0.87 (0.59–1.26)	0.92 (0.63–1.36)	0.77 (0.49–1.22)
GPA	Ref.	0.69 (0.50–0.96) *	0.65 (0.43–0.98) *	0.73 (0.52–1.01)

[a] Based on previous literature [1], participants were considered malnutrition if they met at least three of the five criteria listed below: (1) serum albumin ≤37 g/L, (2) male weight ≤63.9 kg, female weight ≤51.8 kg, (3) serum cholesterol <4.1 mmol/L, (4) energy intake <15 kcal/kg/day, and (5) protein intake <0.5 g/kg/day. Calculated using binary logistic regression models. Model adjusted for age, race/ethnicity, educational level, marital status, PIR, physical activity, alcohol consumption, smoking status, BMI, hypertension, diabetes, arthritis, stroke, gout, cancer, congestive heart failure, coronary heart disease, angina, asthma, chronic bronchitis, emphysema, and total energy intake. 5-Methyltetrahydrofolate: Q1: <28.5 nmol/L for females and <26.1 nmol/L for males; Q2: 28.5–45.1 nmol/L for females and 26.1–38.9 nmol/L for males; Q3: 45.1–71.1 nmol/L for females and 38.9–59.0 nmol/L for males; Q4: ≥71.1 nmol/L for females and ≥59.0 nmol/L for males. Total folate: Q1: <30.7 nmol/L for females and <28.2 nmol/L for males; Q2: 30.7–48.1 nmol/L for females and 28.2–41.4 nmol/L for males; Q3: 48.1–75.5 nmol/L for females and 41.4–62.5 nmol/L for males; Q4: ≥75.5 nmol/L for females and ≥62.5 nmol/L for males. * $p < 0.05$; ** $p < 0.01$.

Table S12. Sensitivity analyses of weighted odds ratios (95% confidence intervals) for functional disability across quartiles of dietary folate intake.

	Quartile of dietary folate intake			
	Q1	Q2	Q3	Q4
LEM	Ref.	1.00 (0.72–1.38)	0.75 (0.54–1.05)	0.76 (0.52–1.12)
IADL	Ref.	1.08 (0.76–1.54)	0.68 (0.50–0.93) *	0.62 (0.45–0.86) **
ADL	Ref.	0.95 (0.60–1.50)	0.79 (0.54–1.14)	0.76 (0.53–1.11)
LSA	Ref.	1.09 (0.75–1.58)	0.90 (0.64–1.25)	1.00 (0.71–1.40)
GPA	Ref.	1.01 (0.71–1.44)	0.72 (0.52–1.01)	0.96 (0.68–1.37)

Calculated using binary logistic regression models. Model adjusted for sex, age, race/ethnicity, educational level, marital status, PIR, physical activity, alcohol consumption, smoking status, BMI, hypertension, diabetes, arthritis, stroke, gout, cancer, congestive heart failure, coronary heart disease, angina, asthma, chronic bronchitis, emphysema, and total energy intake. Q1: <296.5 DFE (mcg); Q2: 296.5–409.5 DFE (mcg); Q3: 409.5–581.5 DFE (mcg); Q4: ≥581.5 DFE (mcg). * $p < 0.05$; ** $p < 0.01$.

Table S13. Sensitivity analyses of the association between folate supplement users and non-supplement users with functional disability.

	Non-supplement users	Supplement users
LEM	Ref.	1.22 (0.96–1.55)
IADL	Ref.	0.82 (0.63–1.08)
ADL	Ref.	0.78 (0.64–0.96) *
LSA	Ref.	0.88 (0.66–1.17)
GPA	Ref.	0.97 (0.77–1.22)

Calculated using binary logistic regression models. Model adjusted for sex, age, race/ethnicity, educational level, marital status, PIR, physical activity, alcohol consumption, smoking status, BMI, hypertension, diabetes, arthritis, stroke, gout, cancer, congestive heart failure, coronary heart disease, angina, asthma, chronic bronchitis, emphysema, and total energy intake. * $p < 0.05$.

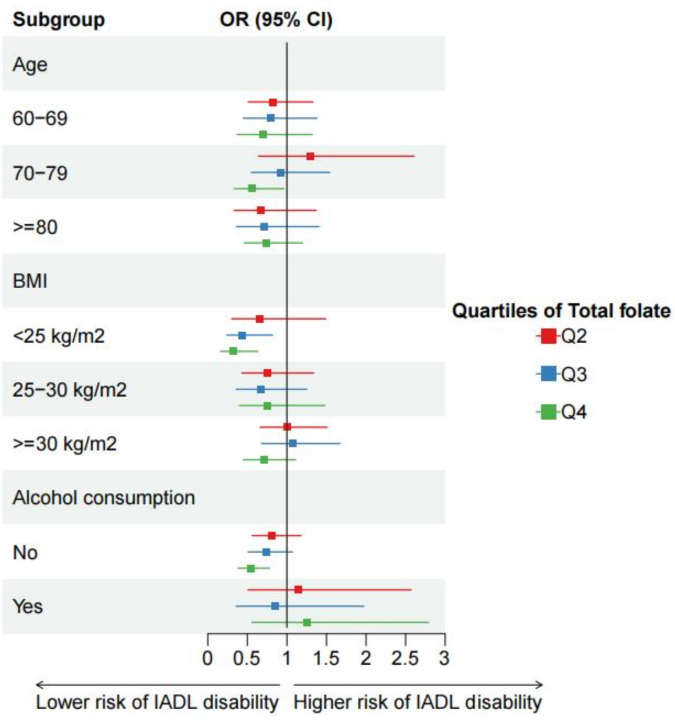

(**a**)

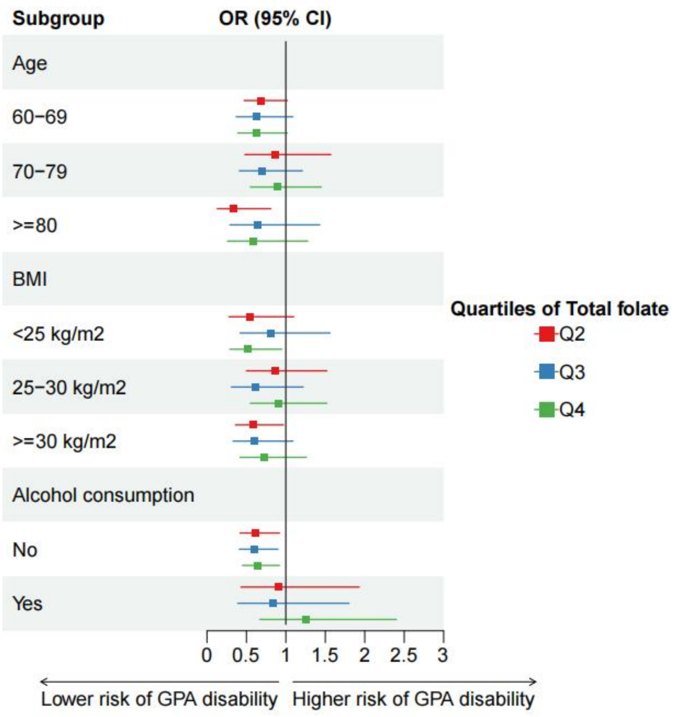

(**b**)

Figure S1. Subgroup analyses of the relationships (multivariate-adjusted odds ratios and 95% confidence intervals) between total folate and the risk of IADL disability (a) and GPA disability (b).

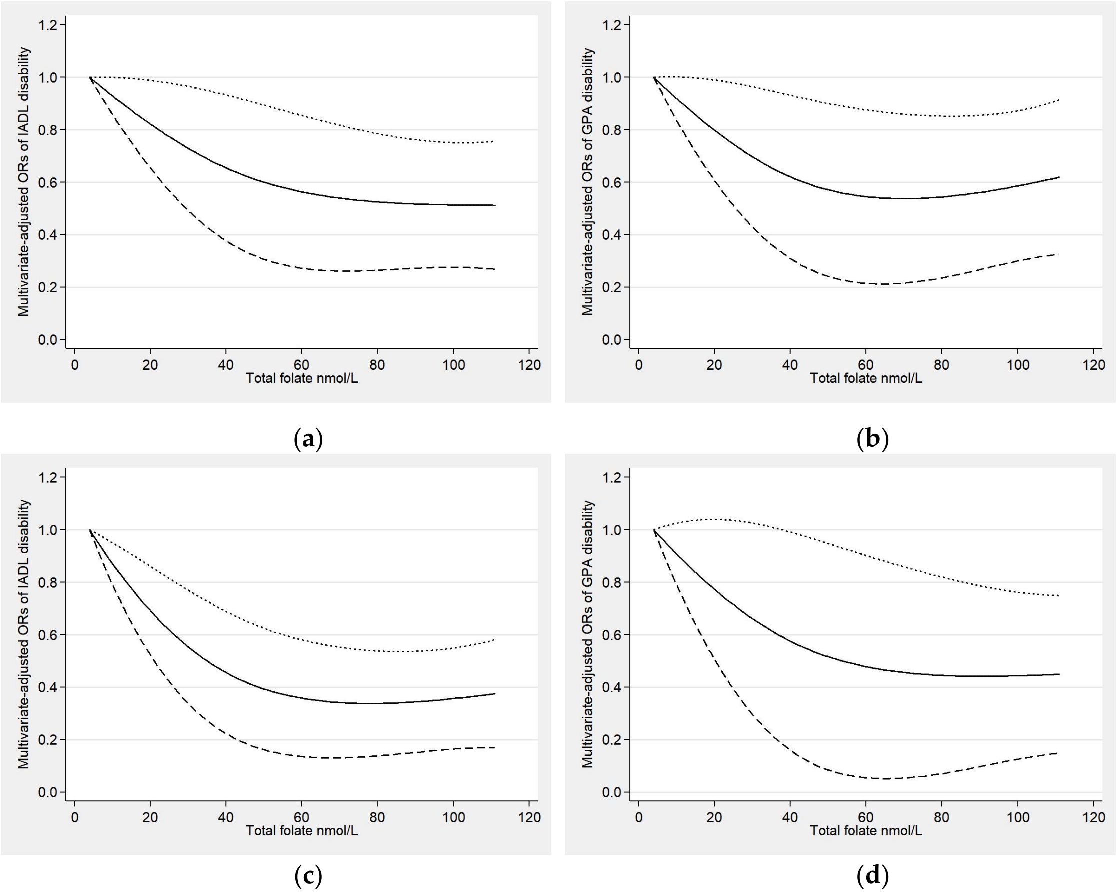

Figure S2. (a) Examination of the dose–response relationship between total folate and IADL disability (p for non-linearity = 0.296); (b) examination of the dose–response relationship between total folate and GPA disability (p for non-linearity = 0.154); (c) examination of the dose–response relationship between total folate and IADL disability in females (p for non-linearity = 0.047); (d) examination of the dose–response relationship between total folate and GPA disability in females (p for non-linearity = 0.345). The solid line and dashed lines represent the estimated odds ratios and the 95% confidence intervals.

Reference

1. Garg, A.X.; Blake, P.G.; Clark, W.F.; Clase, C.M.; Haynes, R.B.; Moist, L.M. Association between renal insufficiency and malnutrition in older adults: results from the NHANES III. *Kidney international* **2001**, *60*, 1867-1874.
